# Supplementary material for: Phytochemical Characterization, and Antioxidant and Antimicrobial Properties of Agitated Cultures of Three Rue Species: Ruta chalepensis, Ruta corsica, and Ruta graveolens
Source: Antioxidants (Basel). 2022 Mar 20;11(3):592. doi: 10.3390/antiox11030592 (PMC8945450; doi:10.3390/antiox11030592)
Supplement: Supplementary file 1 [file antioxidants-11-00592-s001.zip › antioxidants-1631707-supplementary.pdf]

Phytochemical characterization, and antioxidant and antimicrobial properties of agitated cultures of three rue species: *Ruta chalepensis*, *Ruta corsica* and *Ruta graveolens*.

Supplementary files

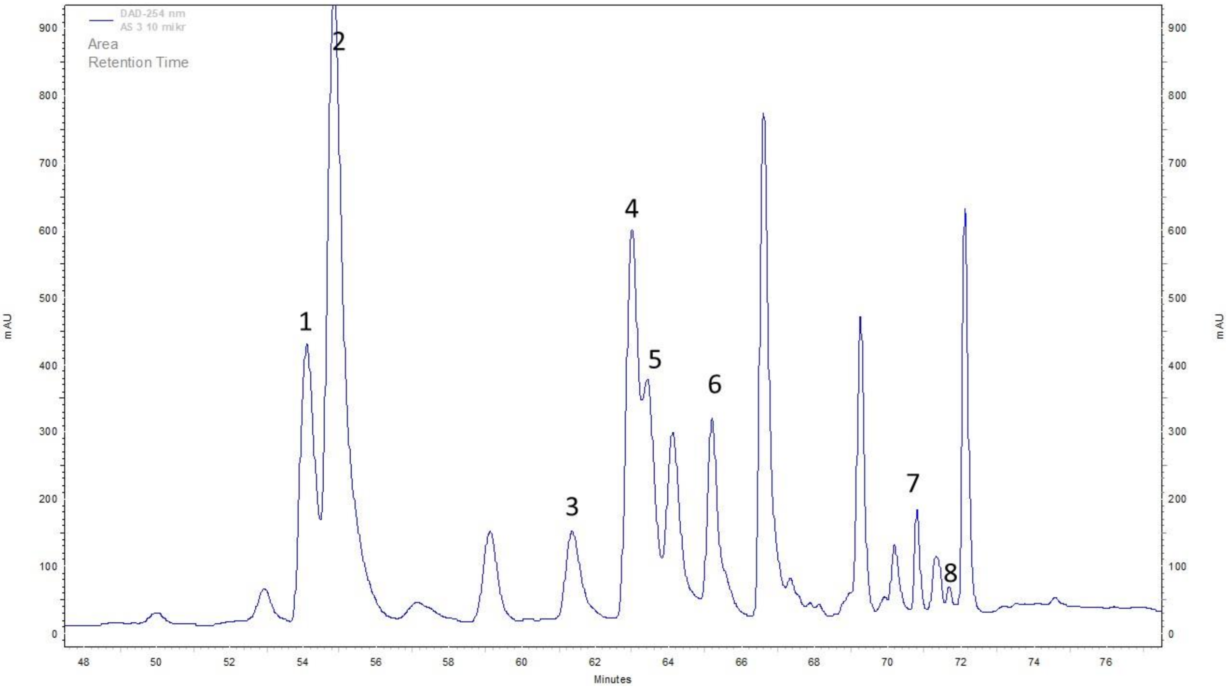

Figure S1 Sample chromatogram of the extract from *Ruta corsica in vitro* cultures (1. psoralen, 2. xanthotoxin, 3. isopimpinellin, 4. skimmianine, 5. bergapten, 6. γ-fagarine, 7. 7-isopentenyloxy-γ-fagarine, 8. isoimperatorin)

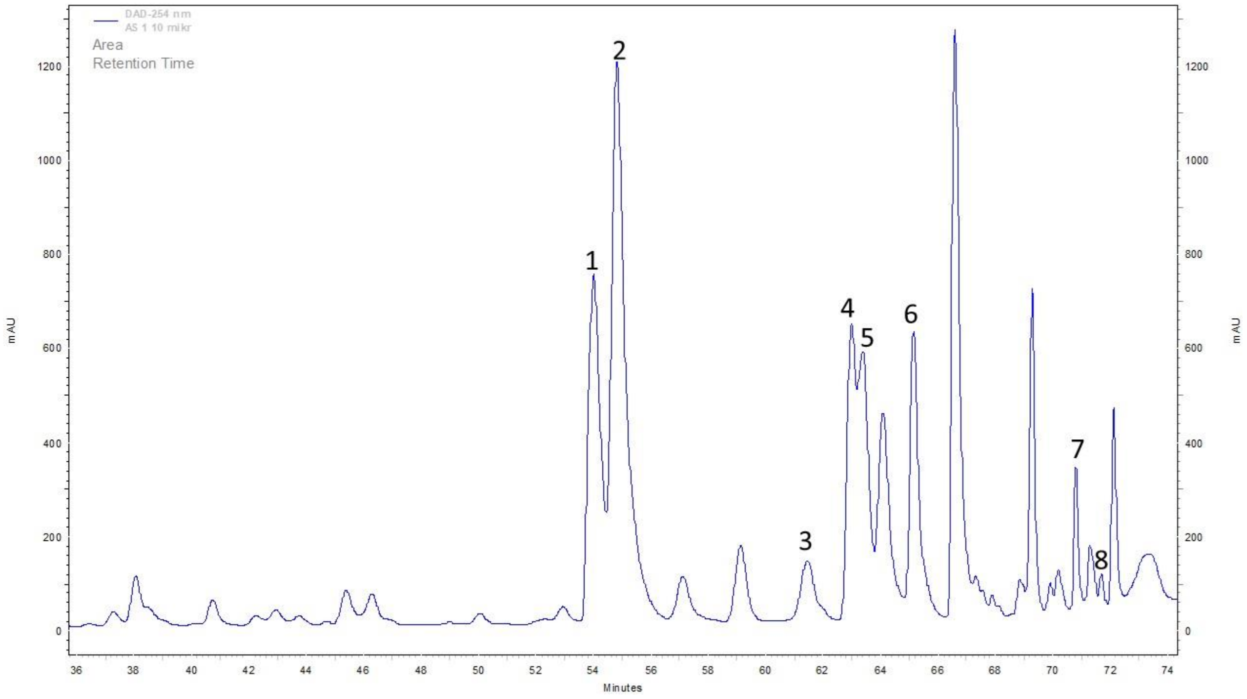

Figure S2 Sample chromatogram of the extract from *Ruta chalepensis in vitro* cultures. (1. psoralen, 2. xanthotoxin, 3. isopimpinellin, 4. skimmianine, 5. bergapten, 6. γ-fagarine, 7. 7-isopentenyloxy-γ-fagarine, 8. isoimperatorin)

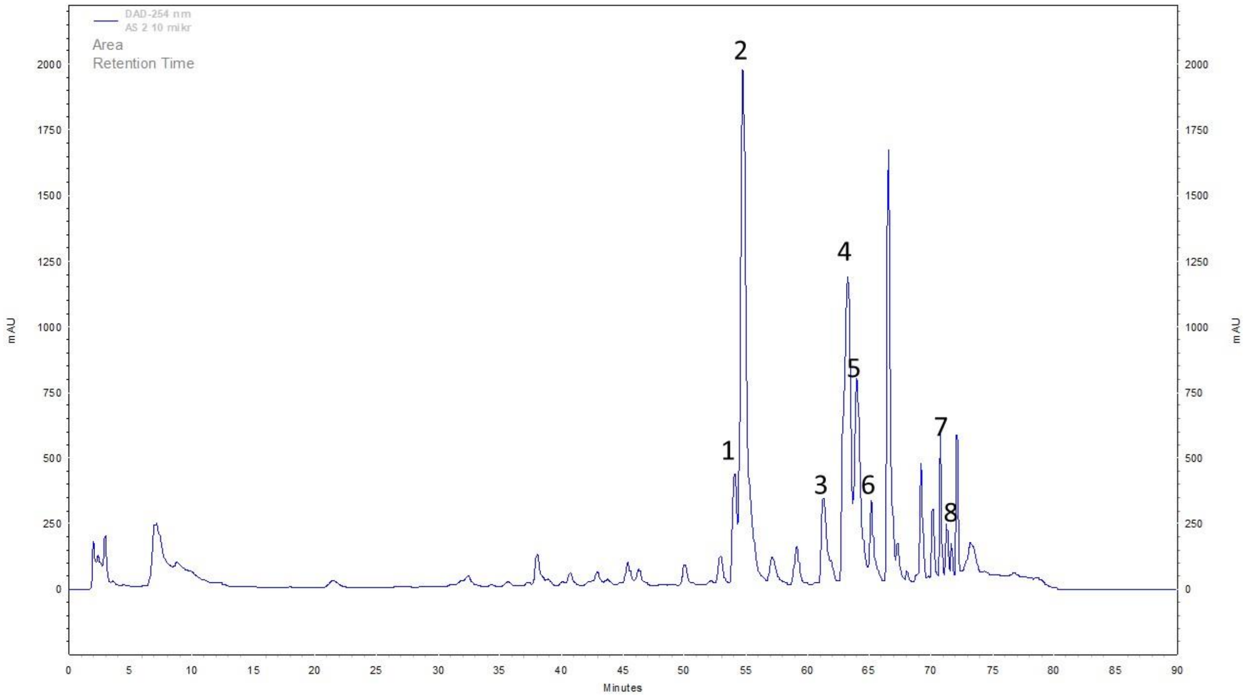

Figure S3 Sample chromatogram of the extract from *Ruta graveolens in vitro* cultures. (1. psoralen, 2. xanthotoxin, 3. isopimpinellin, 4. skimmianine, 5. bergapten, 6. γ-fagarine, 7. 7-isopentenyloxy-γ-fagarine, 8. isoimperatorin)
